# A Comparison of Clinicopathologic Outcomes Across Neoadjuvant and Adjuvant Treatment Modalities in Resectable Gastric Cancer

**DOI:** 10.1001/jamanetworkopen.2021.38432

**Published:** 2021-12-10

**Authors:** Eric Anderson, Alexis LeVee, Sungjin Kim, Katelyn Atkins, Michelle Guan, Veronica Placencio-Hickok, Natalie Moshayedi, Andrew Hendifar, Arsen Osipov, Alexandra Gangi, Miguel Burch, Kevin Waters, May Cho, Samuel Klempner, Joseph Chao, Mitchell Kamrava, Jun Gong

**Affiliations:** 1 Department of Radiation Oncology, Cedars Sinai Medical Center, Los Angeles, California; 2 Division of Hematology and Oncology, Samuel Oschin Comprehensive Cancer Institute, Department of Medicine, Cedars Sinai Medical Center, Los Angeles, California; 3 Biostatistics and Bioinformatics Research Center, Samuel Oschin Comprehensive Cancer Institute, Cedars-Sinai Medical Center, Los Angeles, California; 4 Division of Surgical Oncology, Department of Surgery, Samuel Oschin Comprehensive Cancer Institute, Cedars-Sinai Medical Center, Los Angeles, California; 5 Department of Pathology and Laboratory Medicine, Cedars-Sinai Medical Center, Los Angeles, California; 6 Division of Hematology and Oncology, Department of Medicine, University of California, Irvine, Irvine, California; 7 Department of Medicine, Massachusetts General Hospital Cancer Center, Harvard Medical School, Boston, Massachusetts; 8 Department of Medical Oncology and Therapeutics Research, City of Hope Comprehensive Cancer Center, Duarte, California

## Abstract

**Importance:**

Treatment of resectable gastric cancer (RGC) uses a multimodal approach, including surgical treatment and chemotherapy with or without radiation therapy, and the optimal treatment strategy and timing of each of these modalities is unknown.

**Objective:**

To investigate the association of various neoadjuvant and adjuvant treatment modalities with pathologic complete response (pCR), surgical margin status (SMS), and overall survival (OS) in RGC.

**Design, Setting, and Participants:**

For this comparative effectiveness study, the National Cancer Database was interrogated to identify patients with RGC diagnosed from 2004 to 2015. Patients with gastric adenocarcinoma that was cT2-T4b, any N, and M0 and who underwent definitive surgical treatment were included.

**Main Outcomes and Measures:**

The association of 9 treatment groups (ie, neoadjuvant chemoradiation only [nCRT], neoadjuvant chemotherapy only, adjuvant chemotherapy only [aCT], adjuvant chemoradiation only [aCRT], neoadjuvant chemotherapy and adjuvant radiation, chemotherapy with timing unknown [CTTU], chemoradiation therapy with timing unknown, radiation therapy with timing unknown (RTTU), and no perioperative therapy [NT]) with 3 end points (ie, pCR, SMS, and OS) was analyzed. The analysis was done using logistic regression and Cox proportional hazards models with adjustment for baseline characteristics. Data were analyzed from September 2019 through February 2020.

**Results:**

Among 183 204 patients with RGC who were screened, 3064 patients were included in the analysis (median [IQR] age, 68 [57-77] years; 1764 [57.6%] men). There were 1584 tumors (51.7%) located in the antrum and 1539 stage 2 tumors (50.2%). On multivariable analyses among 1939 patients (owing to 137 patients with missing data for pCR and the exclusion of 988 patients with aCT and aCRT from pCR analysis), nCRT was associated with increased odds of pCR compared with NT, with the greatest odds ratio (OR) among all treatments (OR, 59.55; 95% CI, 10.63-333.56; *P* < .001). RTTU had the next highest OR (29.96; 95% CI, 2.92-307.53; *P* = .004). In multivariable analysis for OS among 3061 patients (owing to missing data for OS), CTTU was associated with decreased risk of death compared with NT (hazard ratio, [HR], 0.41; 95% CI, 0.35-0.48; *P* < .001), with the lowest HR, as was nCRT (HR, 0.48; 95% CI, 0.35-0.66; *P* < .001), with the next lowest HR. Median OS was greatest among patients treated with CTTU (53.9 months; 95% CI, 44.5-61.0 months), followed by nCRT (39.1 months; 95% CI, 26.9 months-not applicable) and aCT (36.1 months; 95% CI, 28.88-49.18 months), while 2-year OS rates were 65.6% (95% CI, 61.3%-69.5%) for CTTU, 63.6% (95% CI, 52.3%-73.0%) for nCRT, and 59.7% (95% CI, 54.2%-64.7%) for aCT.

**Conclusions and Relevance:**

This study found that nCRT was associated with the highest pCR rate, while CTTU (ie, neoadjuvant or adjuvant therapy) was associated with the greatest OS.

## Key Points

**Question:**

Which treatment modality for gastric cancer is associated with the greatest effectiveness?

**Findings:**

In this comparative effectiveness study of 3064 patients with resectable gastric cancer, which compared various treatment regimens of gastric cancer through a modality-by-modality approach, neoadjuvant chemoradiation had the highest pathologic complete response rate, while chemotherapy with timing unknown had the highest survival rate.

**Meaning:**

These findings suggest that neoadjuvant chemoradiation may be superior to alternative treatment modalities for achieving pathologic complete response and that receipt of any chemotherapy may be associated with the greatest survival benefit for patients with resectable gastric cancer.

## Teaser

This comparative effectiveness study assesses the association of various neoadjuvant and adjuvant treatments for resectable gastric cancer with pathologic complete response, surgical margin status, and overall survival.

## Introduction

Treatment of resectable gastric cancer (RGC) uses a multimodal approach, and significantly improving outcomes for patients remains challenging given the aggressive nature of the disease. Randomized clinical trials have found improved progression-free survival and overall survival (OS) when comparing perioperative chemotherapy vs surgical treatment alone,^1,2,3^ adjuvant chemotherapy vs surgical treatment alone,^4,5^ and adjuvant chemoradiotherapy vs surgical treatment alone^6^; however, large head-to-head analyses comparing each of these treatment regimens and combinations have not been performed, to our knowledge. Although perioperative chemotherapy with 5-fluorouricil, leucovorin, oxaliplatin, and docetaxel (FLOT) has become the standard of adjunctive therapy for RGC,^7^ it remains unclear whether alternative combinations of neoadjuvant and adjuvant chemotherapy with or without radiation may be better than perioperative chemotherapy alone.^8,9,10^ Owing to the lack of data regarding the optimal treatment strategy for RGC, there is controversy among major guidelines regarding use and timing of each treatment modality and there is currently no global standard of care.^11,12,13,14^

Accordingly, we evaluated the association of various combinations of neoadjuvant chemotherapy, adjuvant chemotherapy, and radiation with outcomes in the treatment of gastric cancer that was cT2-T4b, any N, and M0. Through a modality-by-modality approach, we compared clinical and pathologic factors for each treatment combination across 3 end points, including pathologic complete response (pCR), surgical margin status (SMS), and OS, to investigate the optimal treatment strategy for RGC.

## Methods

Given that all patient identification variables were removed, Cedars-Sinai Medical Center determined that institutional review board review and informed consent were not needed. The International Society for Pharmacoeconomics and Outcomes Research (ISPOR) reporting guideline was followed by formulating a prespecified research question and analysis and reporting the results in a standardized method.^15^

### Patient Selection

Patients with RGC diagnosed from 2004 to 2015 were found through a deidentified National Cancer Database (NCDB) file (eFigure in the Supplement). Among 183 204 initial patients with gastric cancer, we screened for patients with cancer that was stage cT2-T4b, any N, and M0 and who underwent definitive surgical treatment. Patients who had nonadenocarcinoma histology, M1 or unknown M status, or absent or unknown carbohydrate antigen 19-9 and carcinoembryonic antigen levels were excluded. From the remaining 23 021 patients with RGC, we excluded 17 575 patients because they had no vital status for OS follow-up, unknown surgical treatment status, unknown local therapy status, or unknown chemotherapy administration status. Among the remaining 5446 patients with RGC, we excluded 2382 patients who were missing data to assign to a treatment group (defined in a later section) to reach a final study cohort of 3064 patients with RGC. Owing to missing data for each primary outcome measure, several analyses used smaller sample sizes than the total number of patients with RGC. For analyses of pCR, aCT and aCRT were excluded.

Race and ethnicity classification was determined by the NCDB. Race and ethnicity were assessed to evaluate whether this patient demographic was associated with treatment modality, similar to other comparisons using other demographics, such as age, sex, and income (Table 1). The other race and ethnicity group was based on NCDB classification, and specific groups included and reasons why race and ethnicity were combined were not provided.

**Table 1.** Patient Baseline Demographic, Clinical, and Tumor Characteristics

Variable	Patients, No. (%)										*P* value^a^
	Total (N = 3064)	nCT (n = 641)	nCRT (n = 85)	aCT (n = 353)	aCRT (n = 658)	nCTaRT (n = 112)	CTTU (n = 540)	CRTTU (n = 51)	RTTU (n = 23)	NT (n = 601)	
Age, median (IQR), y	68 (57-77)	63 (53-71)	63 (54-72)	67 (55-76)	64 (54-72)	57 (50-65)	75 (64.5-82)	66 (54-72)	61 (50-71)	76 (66-83)	<.001
Sex											
Women	1300 (42.43)	264 (41.19)	34 (40)	137 (38.81)	278 (42.25)	49 (43.75)	239 (44.26)	17 (33.33)	8 (34.78)	274 (45.59)	.43
Men	1764 (57.57)	377 (58.81)	51 (60)	216 (61.19)	380 (57.75)	63 (56.25)	301 (55.74)	34 (66.67)	15 (65.22)	327 (54.41)	
Race and ethnicity (combined)^b^^,^^c^											
Black	634 (20.69)	126 (19.66)	20 (23.53)	74 (20.96)	161 (24.47)	32 (28.57)	109 (20.19)	10 (19.61)	2 (8.70)	101 (16.81)	<.001
White	1498 (48.89)	308 (48.05)	51 (60)	157 (44.48)	275 (41.79)	42 (37.5)	303 (56.11)	23 (45.1)	8 (34.78)	330 (54.91)	
Other	932 (30.42)	207 (32.29)	14 (16.47)	122 (34.56)	222 (33.74)	38 (33.93)	128 (23.7)	18 (35.29)	13 (56.52)	170 (28.29)	
Insurance type^b^											
Medicaid	274 (8.94)	64 (9.98)	6 (7.06)	34 (9.63)	70 (10.64)	18 (16.07)	30 (5.56)	6 (11.76)	2 (8.70)	43 (7.15)	<.001
Medicare	1650 (53.85)	262 (40.87)	36 (42.35)	179 (50.71)	304 (46.2)	28 (25.00)	382 (70.74)	30 (58.82)	8 (34.78)	421 (70.05)	
Not insured	157 (5.12)	32 (4.99)	3 (3.53)	28 (7.93)	47 (7.14)	8 (7.14)	17 (3.15)	2 (3.92)	2 (8.70)	18 (3.00)	
Other government insurance	30 (0.98)	7 (1.09)	3 (3.53)	2 (0.57)	5 (0.76)	1 (0.89)	6 (1.11)	0	0	5 (0.83)	
Private	953 (31.1)	276 (43.06)	36 (42.35)	111 (31.44)	232 (35.26)	56 (50)	105 (19.44)	13 (25.49)	11 (47.83)	114 (18.97)	
Income, $/y^b^											
<30 000	511 (16.68)	92 (14.35)	9 (10.59)	59 (16.71)	109 (16.57)	19 (16.96)	104 (19.26)	17 (33.33)	3 (13.04)	100 (16.64)	.02
30 000-34 999	481 (15.70)	96 (14.98)	16 (18.82)	53 (15.01)	104 (15.81)	12 (10.71)	76 (14.07)	7 (13.73)	3 (13.04)	115 (19.13)	
35 000-45 999	823 (26.86)	156 (24.34)	23 (27.06)	93 (26.35)	185 (28.12)	41 (36.61)	151 (27.96)	14 (27.45)	6 (26.09)	153 (25.46)	
≥46 000	1248 (40.73)	297 (46.33)	37 (43.53)	149 (42.21)	260 (39.51)	40 (35.71)	209 (38.70)	12 (23.53)	11 (47.83)	233 (38.77)	
Education level, %^b^^,^^d^											
<14	989 (32.29)	239 (37.29)	31 (36.47)	109 (30.88)	188 (28.57)	36 (32.14)	182 (33.7)	9 (17.65)	8 (34.78)	187 (31.11)	.07
14 to <20	629 (20.51)	127 (19.81)	17 (20)	80 (22.66)	134 (20.36)	17 (15.18)	107 (19.81)	11 (21.57)	2 (8.7)	134 (22.3)	
20 to <29	743 (24.24)	148 (23.09)	15 (17.65)	91 (25.78)	174 (26.44)	29 (25.89)	128 (23.7)	9 (17.65)	8 (34.78)	141 (23.46)	
≥29% or more	703 (22.95)	127 (19.81)	21 (24.71)	73 (20.68)	163 (24.77)	29 (25.89)	123 (22.78)	21 (41.18)	5 (21.74)	139 (23.13)	
Treatment site^b^											
Academic	1660 (54.18)	447 (69.73)	53 (62.35)	166 (47.03)	321 (48.78)	71 (63.39)	276 (51.11)	26 (50.98)	10 (43.48)	290 (48.25)	<.001
Nonacademic	1404 (45.82)	194 (30.27)	32 (37.65)	187 (52.97)	337 (51.22)	42 (37.50)	264 (48.89)	25 (49.02)	13 (56.52)	311 (51.75)	
Geographic location^b^											
Midwest	603 (19.68)	124 (19.34)	21 (24.71)	65 (18.41)	128 (19.45)	18 (16.07)	113 (20.93)	5 (9.80)	1 (4.35)	126 (20.97)	<.001
Northeast	879 (28.69)	224 (34.95)	14 (16.47)	90 (25.50)	177 (26.9)	27 (24.11)	157 (29.07)	18 (35.29)	7 (30.43)	165 (27.45)	
South	1042 (34.01)	190 (29.64)	36 (42.35)	131 (37.11)	251 (38.15)	44 (39.29)	173 (32.04)	8 (15.69)	3 (13.04)	205 (34.11)	
West	540 (17.62)	103 (16.07)	13 (15.29)	66 (18.70)	102 (15.5)	23 (20.54)	97 (17.96)	19 (37.25)	11 (47.83)	105 (17.47)	
AJCC T stage^b^											
T2	879 (28.69)	150 (23.40)	18 (21.18)	89 (25.21)	175 (26.60)	26 (23.21)	182 (33.70)	10 (19.61)	3 (13.04)	227 (37.77)	<.001
T3	1493 (48.73)	378 (58.97)	54 (63.53)	166 (47.03)	314 (47.72)	66 (58.93)	241 (44.63)	27 (52.94)	15 (65.22)	233 (38.77)	
T4	692 (22.58)	114 (17.78)	14 (16.47)	99 (28.05)	168 (25.53)	20 (17.86)	118 (21.85)	14 (27.45)	5 (21.74)	141 (23.46)	
Node positivity status^b^											
Negative	1934 (63.12)	286 (44.62)	37 (43.53)	226 (64.02)	413 (62.77)	53 (47.32)	415 (76.85)	31 (60.78)	14 (60.87)	459 (76.37)	<.001
Positive	1130 (36.88)	355 (55.38)	48 (56.47)	127 (35.98)	245 (37.23)	59 (52.68)	125 (23.15)	20 (39.22)	9 (39.13)	142 (23.63)	
Specific location of tumor^b^											
Antrum	1584 (51.7)	292 (45.55)	42 (49.41)	200 (56.66)	337 (51.22)	56 (50.00)	290 (53.70)	31 (60.78)	8 (34.78)	330 (54.91)	<.001
Body	1090 (35.57)	269 (41.97)	32 (37.65)	114 (32.29)	216 (32.83)	43 (38.39)	192 (35.56)	14 (27.45)	12 (52.17)	199 (33.11)	
Fundus	167 (5.45)	48 (7.49)	12 (14.12)	17 (4.82)	24 (3.65)	4 (3.57)	28 (5.19)	6 (11.76)	1 (4.35)	28 (4.66)	
Pylorus	222 (7.25)	32 (4.99)	0	22 (6.23)	81 (12.31)	9 (8.04)	31 (5.74)	0	3 (13.04)	44 (7.32)	
Stage^b^											
1	680 (22.19)	86 (13.42)	8 (9.41)	69 (19.55)	131 (19.91)	18 (16.07)	162 (30.00)	11 (21.57)	3 (13.04)	192 (31.95)	<.001
2	1539 (50.23)	392 (61.15)	51 (60.00)	160 (45.33)	313 (47.57)	58 (51.79)	285 (52.78)	16 (31.37)	11 (47.83)	254 (42.26)	
3	803 (26.21)	161 (25.12)	26 (30.59)	114 (32.29)	202 (30.70)	35 (31.25)	88 (16.30)	22 (43.14)	9 (39.13)	146 (24.29)	
4	42 (1.37)	1 (0.16)	0	10 (2.83)	12 (1.82)	1 (0.89)	5 (0.93)	2 (3.92)	0	9 (1.50)	
CEA level^b^											
High (≥98 ng/mL)	35 (1.14)	11 (1.72)	1 (1.18)	3 (0.85)	4 (0.61)	1 (0.89)	8 (1.48)	0	0	7 (1.16)	.74
Low (<98 ng/mL)	3029 (98.86)	630 (98.28)	84 (98.82)	350 (99.15)	654 (99.39)	111 (99.11)	532 (98.52)	51 (100)	23 (100)	594 (98.84)	
CA 19-9 level^b^											
High (≥98 ng/mL)	341 (11.13)	53 (8.27)	7 (8.24)	62 (17.56)	69 (10.49)	11 (9.82)	60 (11.11)	5 (9.80)	3 (13.04)	70 (11.65)	.40
Low (<97.9 ng/mL)	2723 (88.87)	588 (91.73)	78 (91.76.00)	291 (82.44)	589 (89.51)	101 (90.18)	480 (88.89)	46 (90.20)	20 (86.96)	531 (88.35)	

Abbreviations: aCT, adjuvant chemotherapy only; aCRT, adjuvant chemoradiation only; AJCC, American Joint Committee on Cancer; CA, carbohydrate antigen; CEA, carcinoembryonic antigen; CTTU, chemotherapy with timing unknown; CRTTU, chemoradiation therapy with timing unknown; nCRT, neoadjuvant chemoradiation only; nCT, neoadjuvant chemotherapy only; nCTaRT, neoadjuvant chemotherapy and adjuvant radiation; NT, no perioperative therapy; RTTU, radiation therapy with timing unknown.

SI conversion: To convert CEA level to micrograms per liter, multiply by 1.0.

^a^ *P* value is calculated by Kruskal-Wallis test for age; and χ^2^ test for categorical variables.

^b^ Missing data were imputed by multiple imputation using all variables listed in the table with logistic regression or polytomous logistic regression models as appropriate. χ^2^ statistics are pooled over imputed data.

^c^ The “other” race and ethnicity group was based on National Cancer Database classification, and specific groups included and reasons why race and ethnicity were combined were not provided.

^d^ Percentages are a measure of the number of adults in the patient's zip code who did not graduate from high school and are categorized as equally proportioned quartiles among all US zip codes. This measure of educational attainment for each patient's area of residence is estimated by matching patient zip code recorded at the time of diagnosis against files derived from year 2000 US Census data.

### Treatment Groups and Primary End Points

We stratified our RGC cohort into 9 treatment groups or treatment timeline groups to assess their association with primary end points of pCR, SMS (ie, positive margins), and OS. The following distinct treatment timeline groups were included: neoadjuvant chemoradiation only (nCRT), neoadjuvant chemotherapy only (nCT), adjuvant chemotherapy only (aCT), adjuvant chemoradiation only (aCRT), neoadjuvant chemotherapy and adjuvant radiation (nCTaRT), chemotherapy with timing unknown (CTTU), chemoradiation therapy with timing unknown (CRTTU), radiation therapy with timing unknown (RTTU), and no perioperative therapy (NT). For neoadjuvant therapy–only groups (ie, nCRT and nCT) and adjuvant therapy–only groups (ie, aCT and aCRT), all patients who received chemotherapy with or without radiation therapy prior to or after definitive surgical treatment, respectively, were included irrespective of sequence of modalities (ie, sequenced or concurrent chemotherapy and radiation therapy) and time from surgical procedure. For the nCTaRT subgroup, chemotherapy could have occurred any time prior to surgical treatment (ie, neoadjuvant treatment) and radiation therapy any time after surgical treatment (ie, adjuvant treatment). The subgroups CTTU, CRTTU, and RTTU included patients for whom the NCDB did not clearly specify when that specific treatment modality was given during the treatment course for RGC but stated that the treatment modality was given at some point along with definitive surgical treatment. For example, CTTU comprised patients who received some form of chemotherapy with unspecified timing; therefore, this subgroup may have included patients receiving nCT, aCT, or both (ie, perioperative treatment). The NT subgroup defined patients for whom RGC was treated with a surgical procedure only.

The primary end points were pCR, SMS, and OS, which was calculated from diagnosis to the date of death or censored at last follow-up. The main estimator variable was treatment group.

### Statistical Analysis

Analyses were performed using R statistical software version 4.0.5 (R Project for Statistical Computing) with 2-sided tests at a significance level of *P* = .05. The missing data pattern for variables with missing values was examined using the Little method.^16^ To decrease risk of bias from missing data, missing values were imputed with respect to other variables as estimators using fully conditional specification implemented by the multivariate imputation by chained equations algorithm using the Mice package under the missing-at-random assumption, with 40 imputed data sets corresponding to a maximum loss of efficiency of 5%.^17,18,19^ We analyzed 40 complete data sets separately, and the results were combined using the Rubin formula.^20^ Data were analyzed from September 2019 through February 2020.

Baseline characteristics were compared between treatment groups with Kruskal-Wallis test for age and χ^2^ test or Fisher exact test for categorical variables. Logistic regression models were employed to estimate the association of treatment group with pCR and SMS with and without adjustment for baseline characteristics. Univariate and multivariable analyses of OS were performed using Cox proportional hazards models.^21^ The proportional hazards assumption was assessed with scaled Schoenfeld residuals.^22^ Median follow-up was calculated using the reverse Kaplan-Meier method.^23^ Survival functions were estimated by the Kaplan-Meier method and compared using log-rank tests.^24^ Post hoc pairwise comparisons between treatment timeline groups in their associations with each end point were further performed, and *P* values were adjusted for multiple tests using the Holm procedure.^25^

Multivariable analyses were performed using stepwise variable selection procedures while forcing treatment group into models. The best models were chosen by Akaike information criterion^26^ by selecting the models with the lowest values. Variables included in the final multivariable models were common to all 40 models fitted to 40 imputed data sets. The 40 best models for each end point had 0 to 4 additional variables. Likelihood ratio tests were conducted to compare larger models with the final model, and the results were not statistically significant. Multicollinearity among variables included in the final models was assessed by tolerance and variance inflation factor.

## Results

Among 183 204 patients with RGC screened, 3064 patients were included in the final analysis (eFigure in the Supplement). Patients receiving neoadjuvant therapy specifically included 641 patients receiving nCT, 85 patients receiving nCRT, and 112 patients receiving nCTaRT. Those who received adjuvant therapy only included 353 patients receiving aCT and 658 patients receiving aCRT. Those who received any modality with timing unknown included 540 patients receiving CTTU, 51 patients receiving CRTTU, and 23 patients receiving RTTU (Table 1). There were 601 patients who did not receive any perioperative therapy. Median (IQR) age was 68 (57-77) years, and more than half of patients (1764 [57.6%]) were men. There were 634 Black patients (20.7%), 1498 White patients (48.9%), and 932 patients (30.4%) with other race or ethnicity. More than 50% of patients were treated at academic facilities (1660 patients [54.18%]). Most patients were treated in the South (1042 patients [34.0%]) or Northeast (879 patients [29%]), while 603 patients (19.7%) were treated in the Midwest and 540 patients (17.6%) in the West. Most tumors were located in the antrum (1584 patients [51.7%]) or body (1090 patients [35.6%]), while few were located in the fundus (167 patients [5.5%]) or pylorus (222 patients [7.3%]). Clinical T3 stage was most common (1493 patients [48.7%]), and most patients were node negative (1934 patients [63.1%]). Overall, clinical stage 2 (1539 patients [50.2%]) was most common. There were 35 patients (1.1%) and 341 patients (11.1%) with carcinoembryonic antigen and carbohydrate antigen 19-9 levels of 98 ng/mL or greater (to convert to micrograms per liter, multiply by 1.0), respectively. Patient age, race and ethnicity, insurance type, academic treatment center, geographic location, gastric tumor location, tumor stage, nodal status, and overall staging were each associated with type of treatment regimen received (Table 1).

In logistic regression, multiple parameters were associated with odds of pCR in multivariable analysis among 1939 patients (owing to 137 patients missing data on pCR and the exclusion of 988 patients with aCT and aCRT from this analysis) and in univariate analysis, including T stage, nodal involvement, and treatment group (Table 2). Node positivity, compared with node negativity, was associated with increased odds of pCR in univariate analysis (odds ratio [OR], 3.22; 95% CI, 1.67-6.20; *P* < .001) and multivariable analysis (OR, 2.03; 95% CI, 1.05-3.92; *P* = .04), while T stage was associated with increased odds of pCR in multivariable analysis (OR for T2 vs T4, 3.70; 95% CI, 1.16-11.80; *P* = .03). Treatment group was also associated with odds of pCR. In multivariable analysis, nCRT was associated with increased odds of pCR (OR, 59.55; 95% CI, 10.63-333.56; *P* < .001) compared with NT, as were nCT (OR, 14.60; 95% CI, 2.81-75.73; *P* = .001) and RTTU (OR, 29.94; 95% CI, 2.92-307.01; *P* = .004). In pairwise comparisons of treatment groups among 1939 patients, nCRT remained associated with pCR compared with nCT (OR, 4.08; 95% CI, 1.92-8.69; *P* = .005) and CTTU (OR, 30.78; 95% CI, 7.56-125.30; *P* < .001) (Table 2).

**Table 2.** Univariate and Multivariable Logistic Regression Analyses for Estimators of Pathologic Complete Response

Variable	Patients, No.	Univariate analysis		Multivariable analysis^a^	
		OR (95% CI)	*P* value	OR (95% CI)	*P* value
Treatment timeline group^b^			<.001^c^		<.001^c^
nCRT	78	64.91 (11.59-363.53)	<.001	59.55 (10.63-333.56)	<.001
nCTaRT	110	5.22 (0.54-50.95)	.16	4.96 (0.52-47.32)	.16
CTTU	510	1.87 (0.25-14.26)	.55	1.93 (0.26-14.22)	.52
CRTTU	48	3.93 (0.15-100.27)	.41	4.36 (0.18-106.46)	.37
RTTU	23	25.4 (2.48-260.71)	.007	29.96 (2.92-307.53)	.004
nCT	598	16.25 (3.11-84.95)	.001	14.60 (2.81-75.73)	.001
NT	572	1 [Reference]	NA	1 [Reference]	NA
Comorbidities, No.			.24^c^		NA
1	770	0.55 (0.24-1.25)	.16	NA^d^	NA
≥2	267	0.45 (0.11-1.90)	.28		
0	1890	1 [Reference]	NA		
Grade^e^			.64^c^		NA
2	693	1.57 (0.20-12.10)	.67	NA^d^	NA
3	2070	0.97 (0.13-7.19)	.97		
4	67	1.31 (0.08-21.06)	.85		
1	97	1 [Reference]	NA		
AJCC T stage^e^			.13^c^		.03^c^
T2	837	3.36 (0.98-11.55)	.06	3.70 (1.14-11.99)	.03
T3	1432	2.55 (0.76-8.62)	.13	1.57 (0.51-4.86)	.43
T4	658	1 [Reference]	NA	1 [Reference]	NA
Node positivity^e^					
Positive	1082	3.22 (1.67-6.20)	<.001	2.03 (1.04-3.95)	.04
Negative	1845	1 [Reference]	NA	1 [Reference]	NA
Specific location of tumor^e^			.09^c^		NA
Fundus	159	1.65 (0.78-3.49)	.19	NA^d^	NA
Body	1034	3.44 (1.22-9.69)	.02		
Pylorus	215	0.51 (0.07-3.79)	.51		
Antrum	1519	1 [Reference]	NA		
Stage^e^			.49^c^		NA
2	1477	1.82 (0.71-4.66)	.22	NA^d^	NA
3	764	1.06 (0.34-3.32)	.92		
4	39	0.00 (0.00-NA)	.99		
1	647	1 [Reference]	NA		
CEA level^e^					
High (≥98 ng/mL)	34	0.00 (0.00-NA)	.99	NA^d^	NA
Low (<97.9 ng/mL)	2893	1 [Reference]	NA		
CA 19-9 level^e^					
High (≥98 ng/mL)	329	0.74 (0.16-3.31)	.69	NA^d^	NA
Low (<97.9 ng/mL)	2598	1 [Reference]	NA		

Abbreviations: AJCC, American Joint Committee on Cancer; CA, carbohydrate antigen; CEA, carcinoembryonic antigen; CTTU, chemotherapy with timing unknown; CRTTU, chemoradiation therapy with timing unknown; NA, not applicable; nCRT, neoadjuvant chemoradiation only; nCT, neoadjuvant chemotherapy only; nCTaRT, neoadjuvant chemotherapy and adjuvant radiation; NT, no perioperative therapy; OR, odds ratio; RTTU, radiation therapy with timing unknown.

SI conversion: To convert CEA level to micrograms per liter, multiply by 1.0.

^a^ 1939 observations were used in the multivariable model.

^b^ Firth penalized maximum likelihood estimation was reported to decrease bias in the parameter estimates because quasicomplete separation of data points was observed owing to low pathologic complete response rates in most treatment groups.

^c^ Overall *P* value for a variable with more than 2 categories.

^d^ Dropped out of the model.

^e^ Missing data were imputed by multiple imputation using all variables listed in Table 1 with logistic regression or polytomous logistic regression models as appropriate.

Multiple variables, including tumor stage, grade, and treatment group, were associated with SMS (ie, odds of having positive margins) among 3014 patients in multivariable analysis (owing to missing data for SMS) and in univariate analysis (Table 3). T stage and node positivity were associated with SMS in univariate analysis. Specifically, earlier T stage was associated with decreased odds of SMS (OR for T2 vs T4, 0.51; 95% CI, 0.38-0.70; *P* < .001), while node positivity was associated with increased odds of SMS (OR vs node negativity, 1.27; 95% CI; 1.04-1.56; *P* = .02). In multivariable analysis, overall clinical stage was associated with increased odds of SMS (OR for stage 3 vs stage 1, 1.81; 95% CI, 1.27-2.58; *P* = .001), with higher clinical stages having increased ORs for SMS; for example, stage 4 had a higher OR than stage 3 (OR vs stage 1, 1.94; 95% CI, 0.76-5.00; P = .17), although this outcome was not statistically significant. Pathologic tumor grade was associated with increased odds of SMS in univariate analysis (OR for grade 3 vs 1, 6.57; 95% CI, 2.16-20.05; *P* < .001) and multivariable analysis (OR for grade 3 vs 1, 6.20; 95% CI, 2.02-19.02; *P* = .001). In multivariable analysis, aCT (OR, 1.46; 95% CI, 1.03-2.05; *P* = .032) and nCTaRT (OR, 2.04; 95% CI, 1.27-3.26; *P* = .003) were associated with increased odds of SMS compared with NT. In pairwise comparisons among 3014 patients, aCT (OR, 1.76; 95% CI, 1.25-2.48; *P* = .047) and nCTaRT (OR, 2.46; 8 95% CI, 1.54-3.91; *P* = .006) were associated with increased odds of SMS compared with nCT; no other comparisons reached statistical significance (Table 3).

**Table 3.** Univariate and Multivariable Logistic Regression Analyses for Estimators of Surgical Margin Status

Variable	Patients, No.	Univariate analysis		Multivariable analysis^a^	
		OR (95% CI)	*P* value	OR (95% CI)	*P* value
Treatment timeline group			<.001^b^		.002^b^
nCRT	83	0.78 (0.39-1.55)	.48	0.68 (0.33-1.37)	.28
aCT	345	1.74 (1.25-2.43)	.001	1.46 (1.03-2.05)	.03
aCRT	649	1.31 (0.97-1.76)	.08	1.15 (0.85-1.57)	.36
nCTaRT	111	2.54 (1.61-4.01)	<.001	2.04 (1.27-3.26)	.003
CTTU	532	1.07 (0.78-1.48)	.66	1.15 (0.83-1.59)	.41
CRTTU	49	1.82 (0.92-3.61)	.09	1.44 (0.71-2.92)	.31
RTTU	23	0.64 (0.16-2.47)	.51	0.42 (0.10-1.86)	.26
nCT	631	0.91 (0.67-1.25)	.58	0.83 (0.60-1.15)	.26
NT	591	1 [Reference]	NA	1 [Reference]	NA
Comorbidities, No.			.15^b^		NA
1	798	1.00 (0.81-1.24)	.99	NA^c^	NA
≥2	275	0.70 (0.49-1.01)	.06		
0	1941	1 [Reference]	NA		
Grade^d^			<.001^b^		<.001^b^
2	725	2.87 (0.92-9.00)	.07	2.78 (0.89-8.75)	.08
3	2117	6.57 (2.16-20.05)	<.001	6.20 (2.02-19.02)	.001
4	68	9.49 (2.76-32.59)	<.001	8.58 (2.49-29.63)	<.001
1	104	1 [Reference]	NA	1 [Reference]	NA
AJCC T stage^d^			<.001^b^		
T2	864	0.51 (0.38-0.70)	<.001	NA^c^	NA
T3	1469	0.58 (0.44-0.76)	<.001		
T4	681	1 [Reference]	NA		
Node positivity^d^					
Positive	1114	1.27 (1.04-1.56)	.02	NA^c^	NA
Negative	1900	1 [Reference]	NA		
Specific location of tumor^d^			.35^b^		NA
Fundus	165	0.84 (0.67-1.06)	.15	NA^c^	NA
Body	1074	0.71 (0.41-1.24)	.23		
Pylorus	218	0.99 (0.65-1.51)	.97		
Antrum	1557	1 [Reference]	NA		
Stage^d^			<.001^b^		<.001^b^
2	1516	1.13 (0.83-1.54)	.45	1.10 (0.79-1.53)	.57
3	789	1.92 (1.37-2.69)	<.001	1.81 (1.27-2.58)	.001
4	41	1.94 (0.76-5.00)	.17	1.61 (0.61-4.19)	.33
1	668	1 [Reference]	NA	1 [Reference]	NA
CEA level^d^					
High (≥98 ng/mL)	35	1.19 (0.47-3.00)	.71	NA^c^	NA
Low (<97.9 ng/mL)	2979	1 [Reference]	NA		
CA 19-9 level^d^					
High (≥98 ng/mL)	334	1.22 (0.85-1.75)	.27	1.25 (0.85-1.82)	.25
Low (<97.9 ng/mL)	2680	1 [Reference]	NA	1 [Reference]	NA

Abbreviations: aCT, adjuvant chemotherapy only; aCRT, adjuvant chemoradiation only; AJCC, American Joint Committee on Cancer; CA, carbohydrate antigen; CEA, carcinoembryonic antigen; CTTU, chemotherapy with timing unknown; CRTTU, chemoradiation therapy with timing unknown; NA, not applicable; nCRT, neoadjuvant chemoradiation only; nCT, neoadjuvant chemotherapy only; nCTaRT, neoadjuvant chemotherapy and adjuvant radiation; NT, no perioperative therapy; OR, odds ratio; RTTU, radiation therapy with timing unknown.

SI conversion: To convert CEA level to micrograms per liter, multiply by 1.0.

^a^ 3014 observations were used in the multivariable model.

^b^ Overall *P* value for a variable with more than 2 categories.

^c^ Dropped out of the model.

^d^ Missing data were imputed by multiple imputation using all variables listed in Table 1 with logistic regression or polytomous logistic regression models as appropriate.

In multivariable analysis among 3061 patients (owing to missing data for OS), treatment group was associated with OS, but baseline sociodemographic, clinical, or pathologic variables were not (Figure; Table 4). In multivariable analysis, receipt of nCRT (hazard ratio [HR], 0.48; 95% CI, 0.35-0.66; *P* < .001) and CTTU (HR, 0.41; 95% CI, 0.35-0.48; *P* < .001) were associated with improved OS compared with NT. Adjuvant treatment regimens, including aCT (HR, 0.52; 95% CI, 0.43-0.62; *P* < .001) and aCRT (HR, 0.55; 95% CI, 0.48-63; *P* < .001), were also associated with improved OS compared with NT in multivariable analysis. Neoadjuvant chemotherapy–based approaches, including nCT (HR, 0.61; 95% CI, 0.53-0.71; *P* < .001) and nCTaRT (HR, 0.67; 95% CI, 0.52-0.87; *P* = .003), were also associated with improved OS in multivariable analysis. In pairwise comparisons of treatment groups among 3061 patients, RTTU was associated with worse OS compared with nCT (HR, 2.51; 95% CI, 1.52-4.15; *P* = .008), nCRT (HR, 3.23; 95% CI, 1.81-5.76; *P* = .002), aCT (HR, 3.01; 95% CI, 1.80-5.02; *P* < .001), aCRT (HR, 2.82; 95% CI, 1.71-4.66; *P* = .001), and CTTU (HR, 3.81; 95% CI, 2.30-6.33; *P* < .001). CTTU was associated with improved OS compared with nCT (HR, 0.66; 95% CI, 0.56-0.78; *P* < .001), nCRT (HR, 0.61; 95% CI, 0.46-0.79; *P* = .007), and aCRT only (HR, 0.74; 95% CI, 0.63-0.87; *P* = .009).

**Figure.**
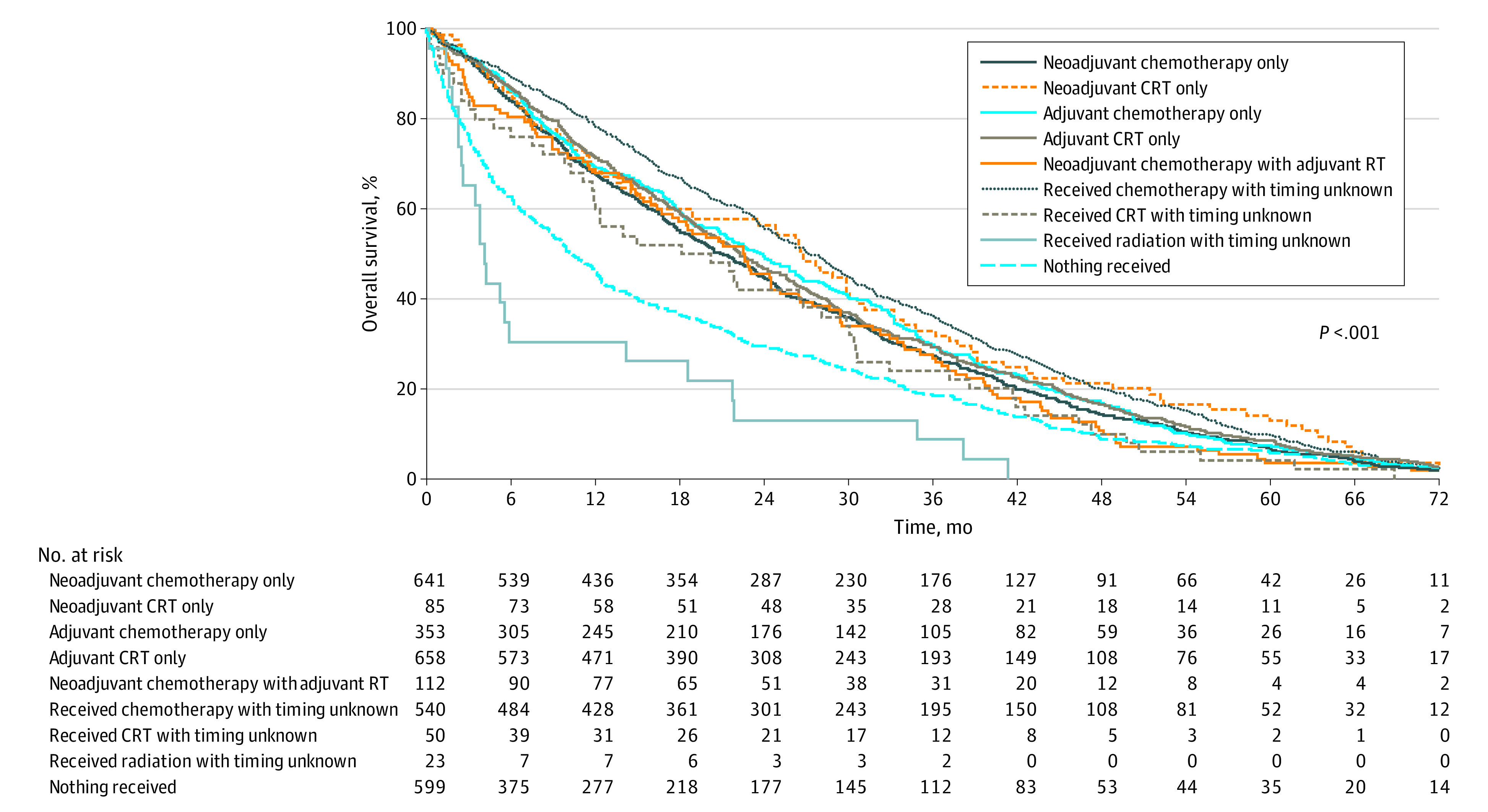
Kaplan-Meier Plot of Overall Survival by Treatment Group Abbreviation: CRT, chemoradiation therapy; RT, radiation therapy.

**Table 4.** Univariate and Multivariable Logistic Regression Analyses for Estimators of Overall Survival

Variable	Patients, No.	Univariate analysis		Multivariable analysis^a^	
		HR (95% CI)	*P* value	HR (95% CI)	*P* value
Treatment timeline group			<.001^b^		<.001^b^
nCRT	85	0.48 (0.35-0.66)	<.001	0.48 (0.35-0.66)	<.001
aCT	353	0.51 (0.43-0.61)	<.001	0.52 (0.43-0.62)	<.001
aCRT	658	0.55 (0.48-0.63)	<.001	0.55 (0.48-0.63)	<.001
nCTaRT	112	0.67 (0.52-0.87)	.002	0.67 (0.52-0.87)	.003
CTTU	540	0.41 (0.35-0.48)	<.001	0.41 (0.35-0.48)	<.001
CRTTU	50	0.62 (0.42-0.92)	.02	0.61 (0.41-0.91)	.016
RTTU	23	1.56 (0.95-2.58)	.08	1.55 (0.94-2.56)	.08
nCT	641	0.61 (0.53-0.71)	<.001	0.62 (0.54-0.71)	<.001
NT	599	1 [Reference]	NA	1 [Reference]	NA
Age, y^c^	3061	1.00 (1.00-1.00)	.60	NA^d^	NA
Sex				NA^d^	NA
Women	1299	1.07 (0.98-1.18)	.15	NA	NA
Men	1762	1 [Reference]	NA		
Race and ethnicity (combined)^e^^,^^f^			.49^b^		NA
Black	632	0.97 (0.86-1.10)	.68	NA^d^	NA
White	1497	1 [Reference]	NA		
Other	932	0.93 (0.84-1.04)	.23		
Insurance type^e^			.51^b^		NA
Medicaid	274	0.98 (0.81-1.17)	.81	NA^d^	NA^d^
Medicare	1647	1.05 (0.94-1.17)	.36		
Other government insurance	30	0.67 (0.37-1.21)	.18		
Not insured	157	1.05 (0.83-1.32)	.69		
Private	953	1 [Reference]	NA		
Income, $/y^e^			.95^b^		NA
30 000-34 999	480	0.99 (0.84-1.18)	.95	NA^d^	NA
35 000-45 999	823	0.99 (0.85-1.15)	.93		
≥46 000	1247	1.03 (0.89-1.18)	.72		
<30 000	511	1 [Reference]	NA		
Education level, %^e^^,^^g^			.51^b^		NA
14-19.9	628	0.95 (0.82-1.09)	.43	NA^d^	NA
20-28.9	743	1.05 (0.93-1.20)	.43		
≥29	701	0.97 (0.85-1.11)	.69		
<14%	989	1 [Reference]	NA		
Treatment site^e^					
Academic (includes integrated network cancer programs)	1659	0.95 (0.86-1.04)	.27	NA^d^	NA
Nonacademic	1402	1 [Reference]	NA		
Geographic location^e^			.21^b^		NA
Midwest	603	0.87 (0.75-1.01)	.06	NA^d^	NA
South	1041	1.00 (0.89-1.13)	.94		
West	539	0.98 (0.85-1.14)	.83		
Northeast	878	1 [Reference]	NA		
Residence area type^e^			.62^b^		NA
Urban	306	0.95 (0.80-1.11)	.51	NA^d^	NA
Rural	44	1.15 (0.78-1.69)	.49		
Metro	2711	1 [Reference]	NA		
Distance between patient residence and hospital, mi^e^			.53^b^		NA
10-20	596	0.95 (0.84-1.08)	.49	NA^d^	NA
>20 to 50	421	0.88 (0.76-1.02)	.09		
>50 to 100	181	0.96 (0.78-1.18)	.71		
>100	122	0.92 (0.72-1.20)	.55		
<10	1741	1 [Reference]	NA		
Year of diagnosis					
2011-2014	2536	0.89 (0.79-1.01)	.07	0.89 (0.79-1.01)	.07
2004-2010	525	1 [Reference]	NA	1 [Reference]	NA
Comorbidities, No.			.54^b^		NA
1	804	1.06 (0.95-1.19)	.27	NA^d^	NA
≥2	277	1.01 (0.85-1.20)	.92		
0	1980	1 [Reference]	NA		
Grade^e^			.45^b^		NA
2	737	0.93 (0.71-1.21)	.58	NA^d^	NA
3	2153	0.87 (0.67-1.12)	.27		
4	68	0.80 (0.52-1.21)	.29		
1	103	1 [Reference]	NA		
AJCC T stage^e^			.76^b^		NA
T2	877	1.01 (0.86-1.20)	.88	NA^d^	NA
T3	1493	1.01 (0.87-1.17)	.94		
T4	691	1 [Reference]	NA		
Node positivity^e^					
Positive	1128	1.00 (0.90-1.11)	.93	NA^d^	NA
Negative	1933	1 [Reference]	NA		
Specific location of tumor^e^			.60^b^		NA
Fundus	166	0.95 (0.75-1.20)	.65	NA^d^	NA
Body	1089	1.06 (0.94-1.19)	.33		
Pylorus	222	0.96 (0.77-1.18)	.67		
Antrum	1584	1 [Reference]	NA		
Stage^e^			.77^b^		NA
2	1537	0.99 (0.85-1.15)	.93	NA^d^	NA
3	802	1.04 (0.88-1.23)	.65		
4	42	0.89 (0.51-1.55)	.68		
1	680	1 [Reference]	NA		
CEA level^e^					
High (≥98 ng/mL)	34	0.72 (0.41-1.26)	.24	NA^d^	NA
Low (<97.9 ng/mL)	3027	1 [Reference]	NA		
CA 19-9 level^e^					
High (≥98 ng/mL)	339	1.05 (0.86-1.28)	.61	NA^d^	NA
Low (<97.9 ng/mL)	2722	1 [Reference]	NA		

Abbreviations: aCT, adjuvant chemotherapy only; aCRT, adjuvant chemoradiation only; AJCC, American Joint Committee on Cancer; CA, carbohydrate antigen; CEA, carcinoembryonic antigen; CTTU, chemotherapy with timing unknown; CRTTU, chemoradiation therapy with timing unknown; HR, hazard ratio; NA, not applicable; nCRT, neoadjuvant chemoradiation only; nCT, neoadjuvant chemotherapy only; nCTaRT, neoadjuvant chemotherapy and adjuvant radiation; NT, no perioperative therapy; RTTU, radiation therapy with timing unknown.

SI conversion: To convert CEA level to micrograms per liter, multiply by 1.0.

^a^ 3061 observations were used in the multivariable model.

^b^ Overall *P* value for a variable with more than 2 categories.

^c^ Hazard ratio for age is expressed as 1-unit increment.

^d^ Dropped out of the model.

^e^ Missing data were imputed by multiple imputation using all variables listed in Table 1 with logistic regression or polytomous logistic regression models as appropriate.

^f^ The other race and ethnicity group was based on National Cancer Database classification, and specific groups included and reasons why race and ethnicity were combined were not provided.

^g^ Percentages are a measure of the number of adults in the patient's zip code who did not graduate from high school and are categorized as equally proportioned quartiles among all US zip codes. This measure of educational attainment for each patient's area of residence is estimated by matching patient zip code recorded at the time of diagnosis against files derived from year 2000 US Census data.

Median OS and estimated 2-year OS rates for treatment groups are presented in the eTable in the Supplement. CTTU had the longest median (IQR) survival, at 53.9 months (95% CI, 44.5-61.0 months), followed by 39.1 months (95% CI, 26.9 months-not applicable) for nCRT and 36.1 months (95% CI, 28.88-49.18 months) for aCT. Treatment with RTTU had the shortest survival (4.2 months; 95% CI, 2.5-18.6 months), including compared with no perioperative treatment (12.3 months among 599 patients [owing to missing data on OS]; 95% CI, 10.4-14.6 months). The 2-year OS rates were 65.6% (95% CI, 61.3%-69.5%) for CTTU, 63.6% (95% CI, 52.3%-73.0%) for nCRT, and 59.7% (95% CI, 54.2%-64.7%) for aCT.

## Discussion

In this comparative effectiveness research study of RGC, we found that nCRT and nCT were associated with increased odds of pCR. This agrees with the results of several phase III clinical trials, including the Medical Research Council Adjuvant Gastric Infusional Chemotherapy (MAGIC),^2^ French Action Clinique Coordonnées en Cancérologie Digestive (ACCORD07),^1^ and Fédération Nationale des Centres de Lutte contre le Cancer (FNCLCC)/Fédération Francophone de Cancérologie Digestive (FFCD) trials,^3^ which found that the addition of perioperative chemotherapy improved surgical outcomes and OS compared with surgical treatment alone. Most recently, the FLOT4 trial^7^ suggested that FLOT should be the new standard of care for perioperative chemotherapy. Given that perioperative therapy improves outcomes and these trials noted difficulty with adherence to adjuvant therapy, the trend in research has shifted toward investigating a purely neoadjuvant approach for RGC. However, phase III trials investigating a purely neoadjuvant approach compared with surgical treatment alone are limited given the benefits previously seen with perioperative chemotherapy. The only phase III trial to investigate a purely neoadjuvant approach compared with surgical treatment alone was the European Organisation for Research and Treatment of Cancer (EORTC) 40954 trial,^27^ which found that neoadjuvant chemotherapy was associated with improved R0 resection rates compared with surgical treatment alone (81.9% vs 66.7%; *P* = .04), but the trial was terminated early owing to poor recruitment. The Phase III Randomized Study of Neoadjuvant Chemotherapy With Docetaxel, Oxaliplatin and S-1 Followed by Surgery and Adjuvant S-1 Vs Surgery and Adjuvant S-1 for Resectable Advanced Gastric Cancer (PRODIGY) trial^28^ is currently being undertaken to investigate neoadjuvant chemotherapy then surgical treatment with adjuvant chemotherapy compared with up-front surgical treatment plus adjuvant chemotherapy. In preliminary data, the addition of neoadjuvant chemotherapy resulted in improved pCR (10.4% vs 0%; *P* < .0001) and progression-free survival (HR, 0.70; 95% CI, 0.52-0.95; *P* = .02) compared with up-front surgical treatment with adjuvant chemotherapy. The association of neoadjuvant treatment with increased rate of pCR found in our study suggests that more research is warranted to investigate neoadjuvant vs perioperative chemotherapy and which population may benefit from a more aggressive up-front approach. Given that adherence to adjuvant chemotherapy was difficult in previous trials, neoadjuvant chemotherapy may be preferred for patients for whom postoperative morbidity is anticipated.

Our study findings also suggest that neoadjuvant chemoradiotherapy may be associated with increased rates of pCR compared with neoadjuvant chemotherapy given that nCRT had increased OR for pCR compared with nCT and pairwise comparisons of nCRT vs nCT for pCR showed a statistically significant difference. The hazard ratio of OS was also decreased in nCRT compared with nCT in univariate and multivariable analyses, suggesting that the increased rates of pCR with nCRT compared with nCT may be associated with improved overall survival. The 2009 phase III Preoperative Chemotherapy vs Chemoradiotherapy in Locally Advanced Adenocarcinomas of the Oesophagogastric Junction (POET) trial^29^ found similar results when comparing pCR rates of neoadjuvant chemotherapy followed by chemoradiotherapy followed by surgical treatment vs neoadjuvant chemotherapy followed by surgical treatment (15.6% vs 2.0%; *P* = .03). This trial also found an increased rate of overall survival for nCRT, but this increase was not statistically significant (HR, 0.65; 95% CI, 0.42-1.01; *P* = .055).^29^ However, the POET trial was limited to patients with cancers of the lower esophagus and gastric cardia; therefore, it is unclear how this can be extrapolated to all gastric cancers.^29^ Retrospective studies comparing nCRT with nCT for gastric cancers, specifically, found similar data regarding improved pCR rates of nCRT compared with nCT, but there were no statistically significant improvements in OS.^30,31^ The phase III Neoadjuvant Trial in Adenocarcinoma of the Esophagus and Esophago-Gastric Junction International Study (Neo-AEGIS)^32^ comparing neoadjuvant carboplatin-paclitaxel plus radiation therapy (CROSS) with perioperative chemotherapy (ie, the MAGIC regimen or FLOT) for locally advanced esophageal and esophago-gastric junction (AEG) cancers is ongoing, and preliminary results found significant differences in pCR for CROSS vs MAGIC-FLOT (16% vs 5%, respectively) but similar 3-year survival rates (56%; 95% CI, 47%-64% vs 57%; 95% CI, 48%-65%). Although this trial examined AEG and esophageal cancers, it begs the question whether pCR is an adequate estimator associated with improved survival for gastric cancer and suggests that perioperative chemotherapy is noninferior to nCRT. The phase III Trial of Preoperative Therapy for Gastric and Esophagogastric Junction Adenocarcinoma (TOPGEAR)^33^ is recruiting patients with RGC specifically to compare nCT with nCRT and may hopefully shed some light on this issue. Adding radiation to chemotherapy in the neoadjuvant setting has several theoretical advantages, including enhancing tumor sensitivity to chemotherapy and improving adherence rates prior to surgical treatment, and can be used palliatively to target symptomatic or bleeding tumors. Further research is warranted to study which patient population may benefit from a more intense approach of incorporating radiation in the neoadjuvant setting.

In our study, when comparing SMS, the receipt of nCRT, nCT, and RTTU had the lowest ORs for having positive surgical margins, although none were statistically significant. Given that these same variables had the highest ORs for pCR, it is not surprising that nCRT, nCT, and RTTU also had the lowest ORs for SMS. We found that nCTaRT and aCT were associated with increased odds of having a surgical margin, which is consistent with positive margins being an indication for adjuvant therapy.^34^

Additionally, we found that CTTU was shown to be the best estimator associated with OS, with the lowest HR for mortality, and had the longest median survival. This finding is not inconsistent with those of the aforementioned studies, which found improved OS with nCT and perioperative chemotherapy, and several phase III trials have found an overall survival benefit for adjuvant chemotherapy compared with surgical treatment alone^4,5^ and adjuvant CRT compared with surgical treatment alone.^6^ The Multicenter Randomized Phase III Trial of Neoadjuvant Chemotherapy Followed by Surgery and Chemotherapy or by Surgery and Chemoradiotherapy in Resectable Gastric Cancer (CRITICS),^35^ which compared preoperative chemotherapy followed by D2 surgical treatment and aCT or aCRT, also found that aCT improved OS compared with aCRT. Our finding that the receipt of CTTU (which includes neoadjuvant, adjuvant, and perioperative chemotherapy) was associated with the greatest OR for survival suggests that chemotherapy should remain as the core pillar in the multimodal approach of RGC treatment.

### Limitations and Strengths

This study has several limitations. The major limitation of this study is selection bias given that treatment regimens were selected for patients based on age, performance status, and tumor characteristics. Adjuvant treatment strategies are also influenced by preoperative treatments and surgical outcomes. Therefore, selected treatment regimens inherently introduce bias because more intense approaches are selected for patients who are medically fit, which may be associated with improved outcomes. In addition, pairwise comparison of OS by treatment group was limited by a small sample size of 23 patients for RTTU. That small sample size was also a limitation in survival time comparisons; however, we recognize that RT alone is not a standard paradigm in the perioperative treatment of RGC with definitive surgical treatment planned. Additionally, the study included only patients who underwent surgical treatment, which may overestimate the association of neoadjuvant treatment with outcomes because individuals who progressed on neoadjuvant treatment were excluded. Another limitation of the study is the retrospective nature of the NCDB, which includes missing variables and incomplete information about treatment regimens and did not allow for standardization of treatment regimens. Despite these limitations, the strengths of this study are the large number of patients included and the comprehensive statistical analysis, which adjusted for multiple variables.

## Conclusions

To our knowledge, this study is the first to compare the effectiveness of the various treatment modalities for RGC by using a modality-by-modality approach. Although we were unable to tease out whether the neoadjuvant or adjuvant portion of patients receiving chemotherapy with timing unknown was associated with the greatest increase in OR for OS, both groups may be associated with OS outcomes given that perioperative FLOT is now widely recognized as a standard in RGC.

This retrospective study, which used a modality-by-modality approach to compare treatments for RGC, found that nCT and nCRT were associated with pCR. We also found that nCRT had increased ORs for pCR compared with nCT. These findings suggest that nCRT may be more effective than nCT in treating tumor burden and may be associated with improved OS. These results support the ongoing TOPGEAR trial, which is investigating the safety and effectiveness of nCRT vs nCT and whether these results lead to differences in OS. Additionally, our study suggests the importance of chemotherapy (including neoadjuvant and adjuvant therapy) as the standard of treatment in the multimodal approach for RGC based on the association of chemotherapy with increased OS.

## Notes

Supplement. **eFigure.** Consolidated Standards of Reporting Trials Diagram of Study Inclusion and Exclusion Criteria **eTable.** Overall Survival by Treatment Group

## References

[1] Boige V, Pignon J, Saint-Aubert B, Final results of a randomized trial comparing preoperative 5-fluorouracil (F)/cisplatin (P) to surgery alone in adenocarcinoma of stomach and lower esophagus (ASLE): FNLCC ACCORD07-FFCD 9703 trial. J Clin Oncol. 2007;25(18_suppl):4510-4510. doi:10.1200/jco.2007.25.18_suppl.4510

[2] Cunningham D, Allum WH, Stenning SP, ; MAGIC Trial Participants. Perioperative chemotherapy versus surgery alone for resectable gastroesophageal cancer. N Engl J Med. 2006;355(1):11-20. doi:10.1056/NEJMoa055531 16822992

[3] Ychou M, Boige V, Pignon JP, . Perioperative chemotherapy compared with surgery alone for resectable gastroesophageal adenocarcinoma: an FNCLCC and FFCD multicenter phase III trial. J Clin Oncol. 2011;29(13):1715-1721. doi:10.1200/JCO.2010.33.0597 21444866

[4] Bang YJ, Kim YW, Yang HK, ; CLASSIC trial investigators. Adjuvant capecitabine and oxaliplatin for gastric cancer after D2 gastrectomy (CLASSIC): a phase 3 open-label, randomised controlled trial. Lancet. 2012;379(9813):315-321. doi:10.1016/S0140-6736(11)61873-4 22226517

[5] Sakuramoto S, Sasako M, Yamaguchi T, ; ACTS-GC Group. Adjuvant chemotherapy for gastric cancer with S-1, an oral fluoropyrimidine. N Engl J Med. 2007;357(18):1810-1820. doi:10.1056/NEJMoa072252 17978289

[6] Macdonald JS, Smalley SR, Benedetti J, . Chemoradiotherapy after surgery compared with surgery alone for adenocarcinoma of the stomach or gastroesophageal junction. N Engl J Med. 2001;345(10):725-730. doi:10.1056/NEJMoa010187 11547741

[7] Al-Batran SE, Homann N, Pauligk C, ; FLOT4-AIO Investigators. Perioperative chemotherapy with fluorouracil plus leucovorin, oxaliplatin, and docetaxel versus fluorouracil or capecitabine plus cisplatin and epirubicin for locally advanced, resectable gastric or gastro-oesophageal junction adenocarcinoma (FLOT4): a randomised, phase 2/3 trial. Lancet. 2019;393(10184):1948-1957. doi:10.1016/S0140-6736(18)32557-1 30982686

[8] Cats A, Jansen EPM, van Grieken NCT, ; CRITICS investigators. Chemotherapy versus chemoradiotherapy after surgery and preoperative chemotherapy for resectable gastric cancer (CRITICS): an international, open-label, randomised phase 3 trial. Lancet Oncol. 2018;19(5):616-628. doi:10.1016/S1470-2045(18)30132-3 29650363

[9] Park SH, Lim DH, Sohn TS, ; ARTIST 2 investigators. A randomized phase III trial comparing adjuvant single-agent S1, S-1 with oxaliplatin, and postoperative chemoradiation with S-1 and oxaliplatin in patients with node-positive gastric cancer after D2 resection: the ARTIST 2 trial. Ann Oncol. 2021;32(3):368-374. doi:10.1016/j.annonc.2020.11.017 33278599

[10] Park SH, Sohn TS, Lee J, . Phase III Trial to compare adjuvant chemotherapy with capecitabine and cisplatin versus concurrent chemoradiotherapy in gastric cancer: final report of the Adjuvant Chemoradiotherapy in Stomach Tumors Trial, including survival and subset analyses. J Clin Oncol. 2015;33(28):3130-3136. doi:10.1200/JCO.2014.58.3930 25559811

[11] National Comprehensive Cancer Network. NCCN guidelines: gastric cancer: version 5.2021. Accessed November 2, 2021. https://www.nccn.org/guidelines/guidelines-detail?category=1&id=1434

[12] Japanese Gastric Cancer Association. Japanese gastric cancer treatment guidelines 2018 (5th edition). Gastric Cancer. 2021;24(1):1-21. doi:10.1007/s10120-020-01042-y 32060757 PMC7790804

[13] Smyth EC, Verheij M, Allum W, Cunningham D, Cervantes A, Arnold D; ESMO Guidelines Committee. Gastric cancer: ESMO clinical practice guidelines for diagnosis, treatment and follow-up. Ann Oncol. 2016;27(suppl 5):v38-v49. doi:10.1093/annonc/mdw350 27664260

[14] Wang FH, Shen L, Li J, . The Chinese Society of Clinical Oncology (CSCO): clinical guidelines for the diagnosis and treatment of gastric cancer. Cancer Commun (Lond). 2019;39(1):10. doi:10.1186/s40880-019-0349-9 30885279 PMC6423835

[15] Berger ML, Mamdani M, Atkins D, Johnson ML. Good research practices for comparative effectiveness research: defining, reporting and interpreting nonrandomized studies of treatment effects using secondary data sources: the ISPOR Good Research Practices for Retrospective Database Analysis Task Force report—part I. Value Health. 2009;12(8):1044-1052. doi:10.1111/j.1524-4733.2009.00600.x 19793072

[16] Little RJA. A test of missing completely at random for multivariate data with missing values. J Am Stat Assoc. 1988;83 (404):1198-1202. doi:10.1080/01621459.1988.10478722

[17] van Buuren S. Multiple imputation of discrete and continuous data by fully conditional specification. Stat Methods Med Res. 2007;16(3):219-242. doi:10.1177/0962280206074463 17621469

[18] van Buuren S, Groothuis-Oudshoorn K. Mice: multivariate imputation by chained equations in R. J Stat Softw. 2011;45(3):1-67. doi:10.18637/jss.v045.i03

[19] White IRR, Royston P, Wood AM. Multiple imputation using chained equations: issues and guidance for practice. Stat Med. 2011;30(4):377-399. doi:10.1002/sim.4067 21225900

[20] Rubin DB. Multiple Imputation for Nonresponse in Surveys. John Wiley & Sons; 1987.

[21] Cox DR. Regression Models and Life Tables. J R Stat Soc Series B Stat Methodol. 1972;34(2):187-220.

[22] Grambsch PM, Therneau TM. Proportional hazards tests and diagnostics based on weighted residuals. Biometrika. 1994;81(3):515-526. doi:10.1093/biomet/81.3.515

[23] Schemper M, Smith TL. A note on quantifying follow-up in studies of failure time. Control Clin Trials. 1996;17(4):343-346. doi:10.1016/0197-2456(96)00075-X 8889347

[24] Kalbfleisch JD, Prentice RL. The statistical analysis of failure time data. Wiley series in probability and mathematical statistics. John Wiley & Sons; 1980.

[25] Holm S. A simple sequentially rejective multiple test procedure. Scand Stat Theory Appl. 1979;6(2):65–70.

[26] Yamashita TY, Yamashita K, Kamumura R. A stepwise AIC method for variable selection in linear regression. Commun Stat Theory Methods. 2007;36(13):2395-2403. doi:10.1080/03610920701215639

[27] Schuhmacher C, Gretschel S, Lordick F, . Neoadjuvant chemotherapy compared with surgery alone for locally advanced cancer of the stomach and cardia: European Organisation for Research and Treatment of Cancer randomized trial 40954. J Clin Oncol. 2010;28(35):5210-5218. doi:10.1200/JCO.2009.26.6114 21060024 PMC3020693

[28] Kang Y, Yook JH, Park Y, Phase III randomized study of neoadjuvant chemotherapy (CT) with docetaxel(D), oxaliplatin(O) and S-1(S) (DOS) followed by surgery and adjuvant S-1, vs surgery and adjuvant S-1, for resectable advanced gastric cancer (GC) (PRODIGY). Ann Oncol. 2019;30(Supplement 5):v851-v934. doi:10.1093/annonc/mdz394.032

[29] Stahl M, Walz MK, Riera-Knorrenschild J, . Preoperative chemotherapy versus chemoradiotherapy in locally advanced adenocarcinomas of the oesophagogastric junction (POET): long-term results of a controlled randomised trial. Eur J Cancer. 2017;81:183-190. doi:10.1016/j.ejca.2017.04.027 28628843

[30] Ikoma N, Das P, Hofstetter W, . Preoperative chemoradiation therapy induces primary-tumor complete response more frequently than chemotherapy alone in gastric cancer: analyses of the National Cancer Database 2006-2014 using propensity score matching. Gastric Cancer. 2018;21(6):1004-1013. doi:10.1007/s10120-018-0832-z 29730720 PMC6515902

[31] Kaltenmeier C, Althans A, Mascara M, . Pathologic complete response following neoadjuvant therapy for gastric adenocarcinoma: a National Cancer Database analysis on incidence, predictors, and outcomes. Am Surg. 2021;87(7):1145-1154. doi:10.1177/0003134820972083 33342268 PMC8213860

[32] Reynolds JV, Preston SR, O’Neill B, Neo-AEGIS (Neoadjuvant trial in Adenocarcinoma of the Esophagus and Esophago-Gastric Junction International Study): preliminary results of phase III RCT of CROSS versus perioperative chemotherapy (modified MAGIC or FLOT protocol). (NCT01726452). J Clin Oncol. 2021;39(15_suppl):4004-4004. doi:10.1200/JCO.2021.39.15_suppl.4004

[33] Leong T, Smithers BM, Haustermans K, . TOPGEAR: A randomized, phase III trial of perioperative ECF chemotherapy with or without preoperative chemoradiation for resectable gastric cancer: interim results from an international, intergroup trial of the AGITG, TROG, EORTC and CCTG. Ann Surg Oncol. 2017;24(8):2252-2258. doi:10.1245/s10434-017-5830-6 28337660

[34] Yang TS, Wang XF, Fairweather M, Sun YH, Mamon HJ, Wang JP. The survival benefit from the addition of radiation to chemotherapy in gastric cancer patients following surgical resection. Clin Oncol (R Coll Radiol). 2020;32(2):110-120. doi:10.1016/j.clon.2019.09.047 31570246

[35] de Steur WO, van Amelsfoort RM, Hartgrink HH, ; CRITICS investigators. Adjuvant chemotherapy is superior to chemoradiation after D2 surgery for gastric cancer in the per-protocol analysis of the randomized CRITICS trial. Ann Oncol. 2021;32(3):360-367. doi:10.1016/j.annonc.2020.11.004 33227408

