# Supplementary material for: A Comparison of Clinicopathologic Outcomes Across Neoadjuvant and Adjuvant Treatment Modalities in Resectable Gastric Cancer
Source: JAMA Netw Open. 2021 Dec 10;4(12):e2138432. doi: 10.1001/jamanetworkopen.2021.38432 (PMC8665367; doi:10.1001/jamanetworkopen.2021.38432)
Supplement: Supplement. — eFigure. Consolidated Standards of Reporting Trials Diagram of Study Inclusion and Exclusion Criteria eTable. Overall Survival by Treatment Group [file jamanetwopen-e2138432-s001.pdf]

Supplemental Online Content

Anderson E, LeVee A, Kim S, et al. A comparison of clinicopathologic outcomes across neoadjuvant and adjuvant treatment modalities in resectable gastric cancer. *JAMA Netw Open*. 2021;4(12):e2138432. doi:10.1001/jamanetworkopen.2021.38432

eFigure. Consolidated Standards of Reporting Trials Diagram of Study Inclusion and Exclusion Criteria

eTable. Overall Survival by Treatment Group

This supplemental material has been provided by the authors to give readers additional information about their work.

© 2021 Anderson E et al. *JAMA Network Open.*

eFigure. Consolidated Standards of Reporting Trials Diagram of Study Inclusion and Exclusion Criteria

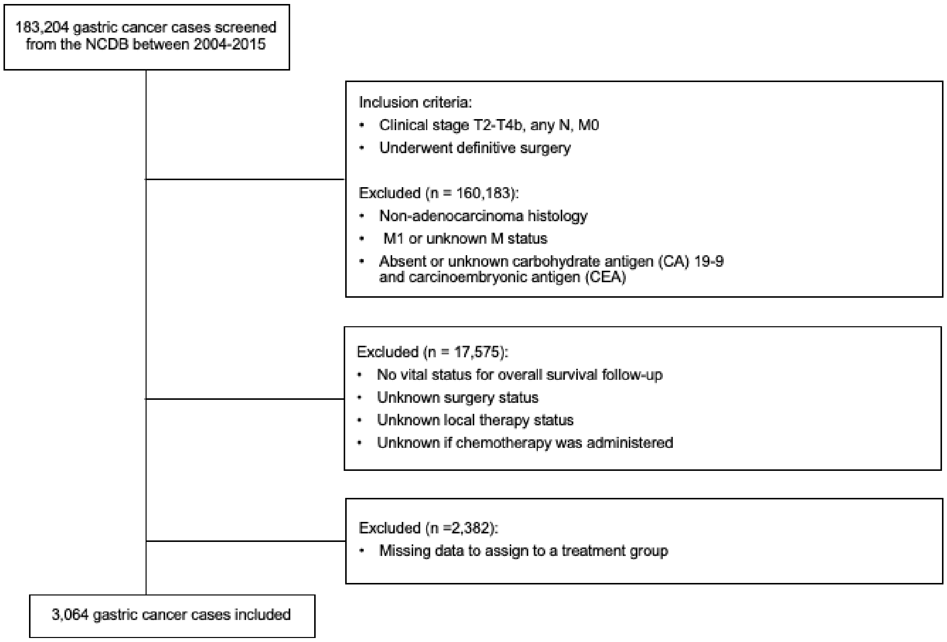

© 2021 Anderson E et al. *JAMA Network Open.*

eTable. Overall Survival by Treatment Group

Treatment groups include neoadjuvant chemoradiation only (nCRT), neoadjuvant chemotherapy only (nCT), adjuvant chemotherapy only (aCT), adjuvant chemoradiation only (acRT), neoadjuvant chemotherapy and adjuvant radiation (nCTaRT), chemotherapy with timing unknown (CTTU), chemoradiation therapy with timing unknown (CRTTU), radiation therapy with timing unknown (RTTU), and no perioperative therapy (NT).

Treatment Timeline groups	No. of Subjects	Median Survival in Months (95% CI)	Estimated 2-year Survival Rate (95% CI)
nCT	641	27.93 (23.59, 31.41)	53.73% (49.67%, 57.61%)
nCRT	85	39.13 (26.87, NA)	63.64% (52.26%, 73.01%)
aCT	353	36.14 (28.88, 49.18)	59.69% (54.21%, 64.74%)
aCRT	658	31.74 (27.1, 40.51)	56.00% (51.99%, 59.81%)
nCTaRT	112	24.51 (17.51, 36.21)	51.85% (42.11%, 60.72%)
CTTU	540	53.88 (44.45, 61.04)	65.60% (61.32%, 69.52%)
CRTTU	50	28.09 (11.96, NA)	52.58% (37.59%, 65.55%)
RTTU	23	4.225 (2.53, 18.6)	29.83% (12.19%, 49.90%)
NT	599	12.32 (10.41, 14.59)	36.84% (32.79%, 40.89%)

© 2021 Anderson E et al. *JAMA Network Open.*
